# Modality Specific Cerebro-Cerebellar Activations in Verbal Working Memory: An fMRI Study

**DOI:** 10.3233/BEN-2010-0266

**Published:** 2010-08-16

**Authors:** Matthew P. Kirschen, S. H. Annabel Chen, John E. Desmond

**Affiliations:** ^1^Department of Radiology and Stanford University School of MedicineStanfordCAUSA; ^2^Neurosciences ProgramStanford University School of MedicineStanfordCAUSA; ^3^Department and Graduate Institute of PsychologyNational Taiwan UniversityTaipeiTaiwan; ^4^Division of PsychologyNanyang Technological UniversitySingapore; ^5^Department of NeurologyJohns Hopkins UniversityBaltimoreMDUSA

**Keywords:** fMRI, cerebellum, modality, verbal working memory, neuroimaging

## Abstract

Verbal working memory (VWM) engages frontal and temporal/parietal circuits subserving the phonological loop, as well as, superior and inferior cerebellar regions which have projections from these neocortical areas. Different cerebro-cerebellar circuits may be engaged for integrating aurally- and visually-presented information for VWM. The present fMRI study investigated load (2, 4, or 6 letters) and modality (auditory and visual) dependent cerebro-cerebellar VWM activation using a Sternberg task. FMRI revealed modality-independent activations in left frontal (BA 6/9/44), insular, cingulate (BA 32), and bilateral inferior parietal/supramarginal (BA 40) regions, as well as in bilateral superior (HVI) and right inferior (HVIII) cerebellar regions. Visual presentation evoked prominent activations in right superior (HVI/CrusI) cerebellum, bilateral occipital (BA19) and left parietal (BA7/40) cortex while auditory presentation showed robust activations predominately in bilateral temporal regions (BA21/22). In the cerebellum, we noted a visual to auditory emphasis of function progressing from superior to inferior and from lateral to medial regions. These results extend our previous findings of fMRI activation in cerebro-cerebellar networks during VWM, and demonstrate both modality dependent commonalities and differences in activations with increasing memory load.

## 1. Introduction

Verbal working memory (VWM) is the temporary storage – and often, the manipulation – of units of linguistic information in memory, allowing the brain to perform higher cognitive functions such as language comprehension and reasoning. Baddeley [2,4,5] proposed a framework for VWM, called the phonological loop, which consists of two components, a phonological short-term store, which can hold speech-related information for 1–2 seconds, and an articulatory control system, which serves to sub-vocally refresh the contents of the phonological store. The manner in which information enters the phonological loop depends upon the modality of presentation: Visually presented stimuli require the rapid conversion of information from a visual to a phonological code, whereas aurally presented stimuli have a more direct route to phonological coding. The present investigation addresses the differences that are observed in brain activation networks underlying verbal working memory when information enters the phonological loop through these different sensory modalities.

Several neuroimaging studies [1] and patient reports [66] have suggested neural correlates for the two

ISSN 0953-4180/10/$27.50 © 2010 – IOS Press and the authors. All rights reserved main components of the phonological loop. The phonological store has been associated with the left inferior parietal regions, whereas the articulatory control system has been functionally linked to the left inferior frontal regions. Desmond and colleagues [23] proposed an extension to Baddeley's VWM framework, in which both the superior and inferior cerebellar hemispheres provide supportive processing to enhance efficiency of neocortical functions through a feed-forward network. Supported by known cerebro-ponto-cerebellar projections from primate studies [11,53], Desmond's model suggests that the superior cerebellum functionally relates to the articulatory control system, while the inferior cerebellum links more closely to the phonological store [15]. Both the cortical and cerebellar regions associated with the phonological loop show increased activation with parametrically increasing memory load [10, 19,29,36,37,52,66]. Our current understanding of how the brain processes VWM has come primarily from studies which assumed that modality specific information translates into amodal phonological codes before rehearsal and retrieval. Thus, the current models of cerebro-cerebellar involvement in VWM have not been fully characterized with stimuli of differing modalities.

Behavioral research has indicated modality differences in memory processing and performance of normal controls suggesting that memory processing in the two modalities is guided by separate streams with different properties and capabilities [46]. However, mixed reports exist as to which modality yields superior performance. While some studies suggested an advantage for auditory stimuli [8,20,40,46], other studies produced an inversion of this modality effect with minor task manipulations [7,47]. Modality-specific effects have also been shown in the inhibitory mechanisms of the central executive component of VWM [43]. Although psychophysical data have not yet provided us with conclusive evidence as to the precise nature of modality specific processing in the brain, it indicates the possibility that auditory and visual information might be processed by separate or distinct but overlapping neural circuits.

Data gathered from brain damaged patients complement and extend the data from healthy controls, further implying that modality specific sensory processing streams exist for the processing of human memory. Studies of brain damaged patients show selective impairments of auditory or visual working memory on a variety of cognitive tasks [6,40,56,57,65,66,69, 70]. For example, Basso et al. [6] described a patient with a left hemisphere lesion who displayed a dissociation between short- and long-term auditory memory, and performed better in the visual input condition. Additionally for left brain-damaged patients, Vallar et al. [65] reported a greater impairment in the recall of phonologically similar than dissimilar stimuli only when the stimuli are presented aurally. Similar deficits have also been described for patients with cerebellar lesions. Silveri et al. [58] reported psychophysical tests of VWM from an 18 year old man after removal of a right cerebellar hemisphere medulloblastoma. The patient demonstrated a phonological-similarity effect for auditory, but not visually presented items, improved memory span with the pointing procedure rather than the verbal response, and the absence of the word-length effect for both modalities with a slight advantage with auditory presentation. This pattern of results suggests that visual and auditory information gain access to the phonological loop via separate pathways. This notion is supported by recent data from our laboratory acquired from children who have undergone cerebellar tumor resection [38]. These children exhibited significantly decreased digit span, relative to control subjects, only when stimuli were presented aurally. Anatomical analyses of lobular damage indicated that damage to left inferior cerebellar hemispheral lobule VIII was highly correlated (after Bonferroni correction) with auditory digit span performance.

The question of whether VWM involves modality specific processing streams has also been addressed in two studies, one employing PET neuroimaging with a 3-back task [55] and the other using fMRI and a 2-back task [21], but results were somewhat conflicting, likely due to differences in memory load and/or imaging modalities. In an n-back task of VWM, Schumacher and colleagues [55] showed highly overlapping brain regions for both auditory and visual memory conditions, and concluded that the frontal-parietal neural circuitry of VWM is amodal. On the other hand, Crottaz-Herbette et al. [21] described important modality differences in addition to similarities in prefrontal and parietal regions. One relevant difference was a tendency for the superior cerebellum to decrease in activation on auditory, but not visual trials. Additional evidence for modality specific processing streams is provided by Ruchkin and colleagues [51], who demonstrated amplitude and timing differences in event-related brain potentials (ERPs) during memory for spoken or written consonant-vowel syllables (non-words). Data indicated that although the phonological loop was activated in both modalities, activation was initiated earlier for aurally presented stimuli, and posterior potentials were larger for visual stimuli.

To date, no published study has employed a Sternberg task to examine modality effects in verbal working memory. Therefore, the goal of the present study is to systematically examine the role of cerebro-cerebellar circuits in the modality specific processing streams of VWM using functional magnetic resonance imaging (fMRI). Data from both healthy subjects [40,61] and brain damaged patients [56,64,65] indicate that auditory information has direct and automatic access to the phonological store of Baddeley's VWM model. Meanwhile, visual information requires phonologic recoding through a rehearsal mechanism before it can be conveyed to the phonological store. Thus, it appears that the articulatory control system has two functions: (1) refreshing phonological traces to keep them active in the phonological store, and (2) translating (or recoding) visual stimuli into phonological representations. Using fMRI, we aim to determine the neural circuits responsible for encoding visually and aurally presented stimuli and facilitating the entry of this information into the phonological loop.

To investigate these modality-dependent effects, we used a task similar to that described by Sternberg [62] and employed in previous studies in our laboratory. As discussed by Braver et al. (1997) we employed a parametric approach to characterize working memory related brain activation in which the control condition used for subtracting out irrelevant processes is a lower load version of (but otherwise identical to) the experimental (higher load) condition. This approach will in theory minimize the likelihood that strategy differences or qualitatively different processes will contaminate the subtraction results, and will instead result in a more pure measure of the process of interest. Results from Chen and Desmond [16] as well as Chein and Fiez [14], showed increased activation in the left frontal region together with the right superior cerebellum during the encoding phase of the task with visual stimuli. Based on these results we reason that if the superior cerebellum contributes to the articulatory process responsible for the orthographic to phonologic recoding of visual information, it will exhibit greater activation for visually presented compared to aurally presented stimuli. Auditory information, on the other hand, with direct access to the phonological loop should not activate the superior cerebellum. Furthermore, based on the data obtained from children with cerebellar tumor removal described above [38], as well as a recent case study report by Chiricozzi and colleagues, we hypothesize that left inferior cerebellum may exhibit more prominent activation when stimuli are delivered aurally [18]. As with other studies, however, we predict that much of the VWM processing will be independent of input modality and thus, there will be a substantial overlap of activity resulting from auditory and visual presentation of information [21,23,55]. Using fMRI, we aim to determine the neural circuits responsible for encoding visually and aurally presented stimuli and facilitating the entry of this information into the phonological loop.

## 2. Materials and methods

### 2.1. Subjects

Subjects were sixteen right-handed subjects (11 male, 5 female) who participated for monetary compensation. All subjects were native speakers of English, with no known psychological or neurological conditions and no history of head trauma. The subjects were on average 21.7 ± 6 years old (± SD). Institutional Review Board approved informed consent was obtained prior to participation in the experiment.

### 2.2. Task procedures

Subjects were instructed to remember 2, 4 or 6 randomly generated consonants (list length) presented at 1 item per second either binaurally or visually in uppercase font (Fig. 1). Sequential presentation was used for visual stimuli to equate timing between the two modality conditions, to ensure subjects were properly encoding the items rather than remembering their placement or orientation and to minimize brain activations associated with eye movements due to scanning or searching an array. Subjects were told to sub-vocally rehearse these letters during a 5 second retention interval and to not use mnemonic or other memory aids. In both the visual and auditory conditions, a lowercase probe letter was then visually presented and subjects indicated with a button press if this probe letter matched a remembered letter in the preceding list (yes – right index finger; no – right middle finger). The probe item was present for the initial 1.5 seconds of a 2 second response interval, followed by an inter-trial-interval (ITI) of 3 seconds. Responses to the probe item were not accepted during the ITI and a failure to make a response did not inhibit the start of the subsequent trial. A fixation cross presented for 1.5 seconds (followed by a 0.5 second delay) indicated the start of each trial. Subjects were instructed to be fast and accurate in their responses.

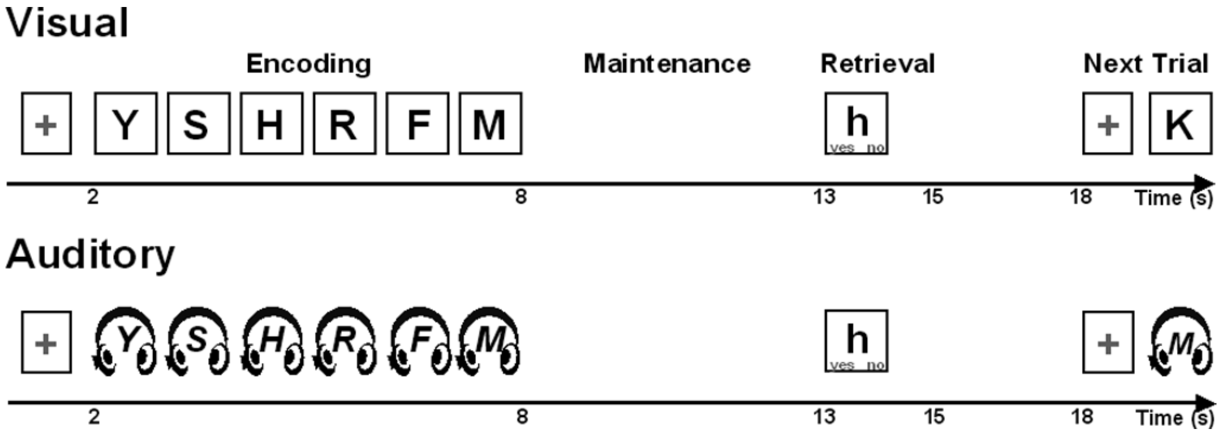

Fig. 1. Task design for investigating modality-dependent VWM activation. Two to six target letters during the encoding phase of the task were presented sequentially in the center of a visual display (visual modality) or binaurally through headphones (auditory modality). Subjects pressed a yes or no button to indicate whether the probe letter matched one of the presented letters.

Both accuracy and reaction time (RT) were collected for each response.

An equal number of targets (items in the presented list) and lures (items not in the presented list) were used as probes. The position of the probe was counterbalanced over all presentation positions. The sequence of list lengths was kept constant across the modality conditions to facilitate direct comparison between the 2 modalities. Each subject completed 4 experimental sessions, two within each modality. The presentation order of the sessions was counterbalanced across subjects. Each session took approximately 10 minutes to complete and consisted of 36 trials, presented in a block design with two trials per block and 6 blocks for each of the three list lengths. Thus, the 2, 4 and 6 letter trials were 12, 14 and 16 seconds long each, and the corresponding block lengths were 24, 28 and 32 seconds, respectively. Subjects practiced for approximately 5 minutes within each modality or until they were comfortable with the task.

Auditory stimuli were presented binaurally through an MR compatible headset and the volume was adjusted appropriately for each subject. Stimuli were created through and driven by Matlab (Mathworks, Natick, MA) using the Psychophysics Toolbox extensions [9, 45] on an Apple Macintosh G3 computer (Apple Computer, Cupertino, CA) and displayed visually with an MR compatible LCD projector (Resonance Technology, Van Nuys, CA). An MR compatible keypad (Resonance Technology, Van Nuys, CA) collected responses.

### 2.3. MRI data acquisition

All MRI data were acquired on a GE 3.0T whole body scanner (General Electric Medical Systems Signa, Waukesha, WI) equipped with a transmit/receive quadrature endcap birdcage resonator head coil. Sufficient padding around the head minimized head movement during the scanning session.

#### 2.3.1. Structural MRI protocol

30 coronal slices of T2-weighted fast spin echo images (TR = 4000, TE = 85, echo train length = 8) were collected to cover the whole brain, with slice thickness of 6 mm. This acquisition was used for anatomical coregistration with the functional volumes.

#### 2.3.2. Functional MRI protocol fMRI scanning was performed with a single-interleave T2*-weighted gradient echo spiral in/out pulse sequence [28] (TR = 2000 ms, TE = 30 ms, flip = 75 degrees, field of view 24 cm). Whole brain functional scans (30 slices) were collected in the coronal plane with an in-plane spatial resolution of 3.75 mm and 6 mm slice thickness at 2 seconds per image. The scan was initiated automatically from the Matlab stimulus presentation script. Subjects were reminded of the task instructions and prompted that the session was about to begin while lying within the scanner.

### 2.4. Data analysis

#### 2.4.1. Behavioral data analysis

Reaction time and accuracy data were recorded for each subject. A repeated measures analysis of variance (ANOVA) tested effects of list length and session on memory performance. Subjects responded with high accuracy across all loads. The analyses excluded reaction time data for incorrect or unanswered trials.

#### 2.4.2. Imaging data analysis

Standard image reconstruction, preprocessing and statistical analyses were performed using the Statistical Parametric Mapping (SPM99) software package (Wellcome Department of Cognitive Neurology, London, UK). The images were realigned and resliced for motion correction, and the structural image was coregistered to the mean motion-corrected functional image for each subject. The functional and structural images were then put into a common coordinate system by normalizing them to the SPM99 template in Montreal Neurological Institute (MNI) space using a twelve-parameter affine normalization routine, and the volumes were smoothed with a Gaussian kernel of 5 mm (FWHM). A general linear model approach was used to analyze individual subject activations [27], as implemented in SPM99. A t-value at each voxel tested contrasts between conditions. A random effects analysis was used to compute the average load response for all 16 subjects. To perform this analysis, one image per contrast, collapsed over the duration of the experiment, was calculated for each subject. Linear trends for both modalities were computed across the load conditions for the whole brain. Conjunction analyses were then computed to identify voxels with (a) significant visual response; (b) significant auditory response; or (c) significant visual and auditory responses (Fig. 3). In computing each conjunction, thresholds of $p < 0.001$ were set for the linear contrasts to define visual- and auditory-responsive voxels, and all voxels survived a false discovery rate correction for multiple comparisons ($p < 0.05$). Linear components were extracted using contrasts of [$-1$ 0 1] for the load 2, 4 and 6 conditions. Additionally, differences between modalities were tested by examining the modality x load interaction. Activated voxels which differed between the modalities were thresholded at $p < 0.005$ (uncorrected, Table 2).

Coordinates depicted in Tables 1 and 2 were transformed from MNI into the coordinate system of the Talairach and Tourneaux stereotaxic atlas [63] using the MNI2TAL transformation described by Lancaster et al. [39]. Anatomical locations corresponding to Talairach coordinates were obtained from the Talairach and Tourneaux atlas [63] for neocortical regions of activation, and from the atlas of Schmahmann et al. [54] for cerebellar regions of activation. Custom-written software was used to display the activation maps coregistered on the normalized T2-weighted anatomy scans [24]. Activation maps are presented on a normalized T1-weighted scan.

## 3. Results

### 3.1. Behavioral performance

There was an overall significant increase in RT with increasing memory load (F(2,30) = 86.46, $p < 0.001$) and a marginal advantage for visual stimuli (F(1,15) = 4.27, $p = 0.06$) (Fig. 2). The memory load by modality interaction was not significant (F(2,30) = 0.92, NS). The parametric increase in memory load had a highly significant linear component (F(1,15) = 183.66, $p < 0.001$) and a non-significant quadratic trend (F(1,15) = 0.31, NS). Only correct responses were included in these behavioral analyses of latency. Subject responses were significantly more accurate with fewer memory items (F(2,30) = 17.86, $p < 0.001$) and demonstrated better accuracy in the visual modality (F(1,15) = 138.41, $p < 0.001$) (Fig. 2). There was no significant interaction effect between memory load and modality (F(2,30) = 0.57, NS). Importantly, for the purpose of comparing brain activations, there was no change in accuracy ($t = -0.74$, NS) or reaction time ($t = -1.31$, NS) between modalities when comparing the high load (6 item) vs. low load (2 item) conditions.

### 3.2. Functional brain activations

Table 1 presents the maximum standard locations of activations for the linear contrast in both auditory and visual modalities at a threshold of $p < 0.001$ (uncorrected). Figure 3 displays regions which activated only during either auditory (green) or visual (red) VWM, as well as areas which responded during both modalities (yellow). Regions in this figure are the result of a conjunction analysis between auditory and visual modalities and represent the overlap of the individual activation maps.

Activations common to both modalities in the left cerebral hemisphere were observed in the cingulate gyrus (Fig. 3), insular cortex, inferior and middle frontal gyri, precentral gyrus, and the inferior parietal lobule/supramarginal gyrus. In the right cerebral hemisphere, common areas of activation included the cingulate gyrus, inferior and middle frontal gyri, precentral gyrus, and inferior parietal and angular gyri. The cerebellum showed overlapping activations in both left and right superior regions (lobule VI) and in right inferior regions (lobules VIIIB and VIIIA). Regions integral to auditory processing, specifically the bilateral temporal gyri, were exclusively activated during auditory VWM, while visual specific activations were observed in the

Table 1
Activations for high vs low memory load to aurally and visually presented stimuli

| Hem | SPM{Z} | x | y | z | Location | Descrip | Nvox | Descrip | Location | x | y | z | SPM{Z} |
|---|---|---|---|---|---|---|---|---|---|---|---|---|---|
| **Auditory and Visual Common Areas of Activation** | | | | | | | | | | | | | |
| Left | 4.9 | -59 | 7 | 17 | Inf Fr Gyr | BA 44 | 924 | BA 6 | Prec Gyr | -50 | -6 | 33 | 5.5 |
| Left | 4.9 | -51 | -8 | 35 | Prec Gyr | BA 6 | | BA 44 | Inf Fr Gyr | -57 | 7 | 15 | 5 |
| Left | 5.1 | -23 | -57 | -29 | Sup Cbl | HVI | 351 | Sup Cbl | HVI | -23 | -57 | -30 | 4.9 |
| Left | 5.7 | -9 | 10 | 41 | Cing Gyr | BA 32 | 306 | BA 6 | Med Fr Gyr | -7 | -5 | 54 | 5.9 |
| Left | 5.2 | -32 | 21 | 4 | Insula | BA 13 | 180 | BA 45 | Inf Fr Gyr | -31 | 25 | 4 | 4.1 |
| Left | 4.0 | -37 | -48 | 33 | Supram Gyr | BA 40 | 108 | BA 40 | Inf Par Lob | -35 | -50 | 35 | 5.8 |
| Left | 4.5 | -64 | -27 | -1 | Mid Temp Gyr | BA 21 | 16 | BA 22 | Sup Temp Gyr | -64 | -24 | 1 | 3.5 |
| Right | 4.7 | 29 | -53 | -33 | Sup Cbl | HVI | 348 | HVI | Sup Cbl | 18 | -61 | -28 | 5.2 |
| Right | 5.2 | 6 | 20 | 35 | Cing Gyr | BA 32 | 192 | BA 32 | Cing Gyr | 6 | 14 | 38 | 4.8 |
| Right | 4.8 | 32 | 30 | 29 | Mid Fr Gyr | BA 9 | 137 | BA 9 | Mid Fr Gyr | 38 | 34 | 26 | 3.8 |
| Right | 4.6 | 29 | 18 | 8 | Claustrum | | 127 | | Insula | 31 | 19 | 3 | 4.1 |
| Right | 4.3 | 50 | -11 | 38 | Prec Gyr | BA 4 | 114 | BA 4 | Prec Gyr | 50 | -9 | 42 | 4.2 |
| Right | 4.5 | 43 | -45 | 40 | Inf Par Lob | BA 40 | 85 | BA 40 | Inf Par Lob | 41 | -50 | 36 | 4.4 |
| Right | 4.3 | 20 | -56 | -53 | Inf Cbl | HVIIIA/B | 69 | HVIIIA | Inf Cbl | 22 | -60 | -55 | 4.1 |
| Right | 4.1 | 30 | -56 | 39 | Angular Gyr | BA 39 | 41 | BA 39 | Angular Gyr | 30 | -54 | 36 | 3.9 |
| Right | 3.7 | 47 | -4 | 26 | Prec Gyr | BA 6 | 40 | BA 6 | Prec Gyr | 43 | 2 | 27 | 3.7 |
| **Auditory Only** | | | | | | | | | | | | | |
| Left | 5.3 | -58 | -5 | -3 | Mid Temp Gyr | BA 21 | 1082 | | | | | | |
| Left | 4.4 | -59 | -32 | 4 | Mid Temp Gyr | BA 22 | | | | | | | |
| Left | 4.3 | -60 | -24 | -1 | Sup Temp Gyr | BA 21 | | | | | | | |
| Left | 4.6 | -21 | -51 | -50 | Inf Cbl | HVIIIB | 341 | | | | | | |
| Left | 4.5 | -28 | -55 | -48 | Inf Cbl | HVIIIA | | | | | | | |
| Left | 5.4 | -34 | 21 | 4 | Insula | BA 13 | 262 | | | | | | |
| Left | 4.8 | -38 | -53 | -32 | Sup Cbl | Crus I | 236 | | | | | | |
| Left | 4.2 | -23 | -59 | -23 | Sup Cbl | HVI | | | | | | | |
| Left | 4.6 | -10 | 22 | 26 | Ant Cing | BA 32 | 145 | | | | | | |
| Left | 4.6 | -9 | 14 | 33 | Cing Gyr | BA 24 | | | | | | | |
| Left | 3.9 | -49 | 6 | 22 | Inf Fr Gyr | BA 44 | 125 | | | | | | |
| Left | 3.8 | -46 | 3 | 15 | Inf Fr Gyr | BA 44 | | | | | | | |
| Left | 3.5 | -46 | -44 | 39 | Inf Par Lob | BA 40 | 33 | | | | | | |
| Left | 3.7 | -28 | -6 | -24 | Uncus | Amyg | 24 | | | | | | |
| Left | 3.5 | -33 | -31 | -3 | Sub-Gyral | Hipp | 13 | | | | | | |
| Right | 4.7 | 54 | -30 | 2 | Mid Temp Gyr | BA 21 | 578 | | | | | | |
| Right | 4.5 | 53 | -20 | 5 | Sup Temp Gyr | BA 41 | | | | | | | |
| Right | 4.8 | 29 | 22 | 14 | Insula | BA 13 | 184 | | | | | | |
| Right | 4.6 | 6 | 22 | 30 | Cing Gyr | BA 32 | 176 | | | | | | |
| Right | 4.6 | 20 | -53 | -51 | Inf Cbl | HVIIIB | 157 | | | | | | |
| Right | 4.3 | 52 | 0 | 23 | Inf Fr Gyr | BA 9 | 136 | | | | | | |
| Right | 4.1 | 47 | -13 | 36 | Prec Gyr | BA 4 | | | | | | | |
| Right | 4.0 | 54 | -7 | 37 | Prec Gyr | BA 6 | | | | | | | |
| Right | 3.9 | 38 | 30 | 22 | Mid Fr Gyr | BA 46 | 74 | | | | | | |
| Right | 5.2 | 32 | 29 | 31 | Mid Fr Gyr | BA 9 | 62 | | | | | | |
| Right | 4.2 | 49 | -16 | -23 | Sub-Gyral | BA 20 | 53 | | | | | | |
| Right | 4.2 | 4 | 1 | 53 | Med Fr Gyr | BA 6 | 36 | | | | | | |
| Right | 3.5 | 39 | -47 | 47 | Inf Par Lob | BA 40 | 36 | | | | | | |
| Right | 3.4 | 43 | -66 | 44 | Inf Par Lob | BA 7 | 33 | | | | | | |
| | | | | | | | | **Visual Only** | | | | | |
| Left | | | | | | | 945 | BA 37 | Fusiform Gyr | -44 | -60 | -15 | 4.8 |
| Left | | | | | | | 332 | BA 40 | Inf Par Lob | -33 | -54 | 35 | 5.1 |
| Left | | | | | | | | BA 19 | Cuneus | -28 | -74 | 27 | 4.6 |
| Left | | | | | | | 102 | BA 6 | Prec Gyr | -39 | -5 | 54 | 4.8 |
| Left | | | | | | | 88 | BA 9 | Inf Fr Gyr | -46 | 3 | 29 | 4.7 |
| Left | | | | | | | 37 | Cbl | Crus I/HVIIB | -41 | -37 | -36 | 4.7 |
| Right | | | | | | | 527 | BA 19 | Inf Occ Gyr | 38 | -69 | -7 | 4.9 |
| Right | | | | | | | 513 | Inf Cbl | HVIIB | 33 | -47 | -39 | 5.1 |
| Right | | | | | | | | Sup Cbl | Crus I | 34 | -60 | -31 | 4.7 |
| Right | | | | | | | | Sup Cbl | HVI/Crus I | 25 | -64 | -32 | 4.6 |
| Right | | | | | | | 163 | BA 40 | Inf Par Lob | 39 | -52 | 38 | 3.8 |
| Right | | | | | | | | BA 40 | Inf Par Lob | 32 | -50 | 36 | 3.6 |
| Right | | | | | | | 57 | Inf Cbl | HVIIIA | 22 | -62 | -53 | 3.8 |
| Right | | | | | | | 50 | BA 40 | Inf Par Lob | 41 | -37 | 39 | 4.0 |
| Right | | | | | | | 39 | BA 9 | Inf Fr Gyr | 41 | 4 | 23 | 4.1 |

Activations thresholded at $p < 0.001$ and voxels are 2 mm$^3$. Entries without the number of activated voxels indicated denote local maxima within a cluster. Abbreviations: Ant = Anterior, BA = Brodmann Area, Cbl = Cerebellum, Cing = Cingulate, Descrip = Description, Fr = Frontal, Gyr = Gyrus, Hem = Hemisphere, Inf = Inferior, Lob = Lobule, Med = Medial, Mid = Middle, Nvox = Number of voxels, Occ = Occipital, Par = Parietal, Prec = Precentral, Sup = Superior, Supram = Supramarginal, Temp = Temporal.

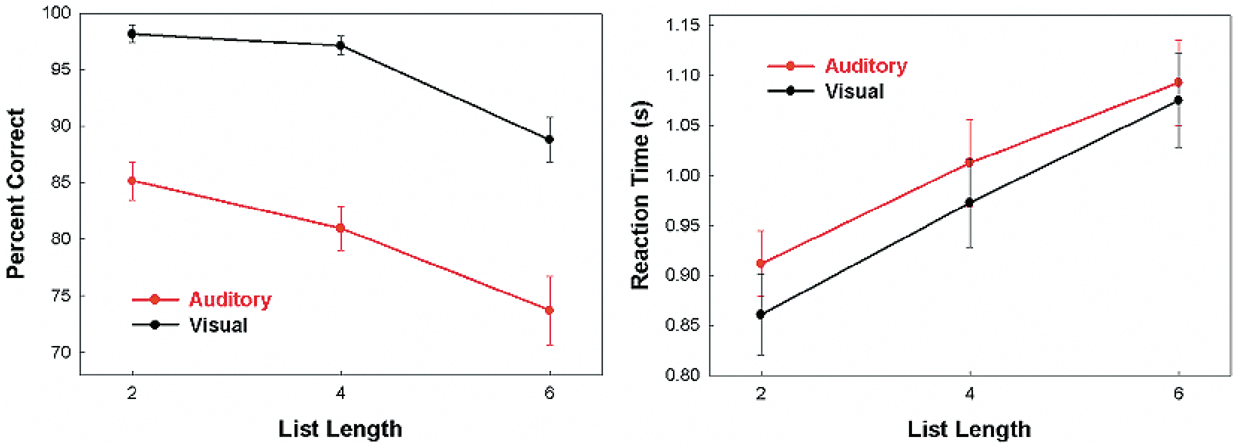

Fig. 2. Reaction time and accuracy plots. Plot of reaction time (left) vs. memory load (list length) and accuracy (right) vs. memory load.

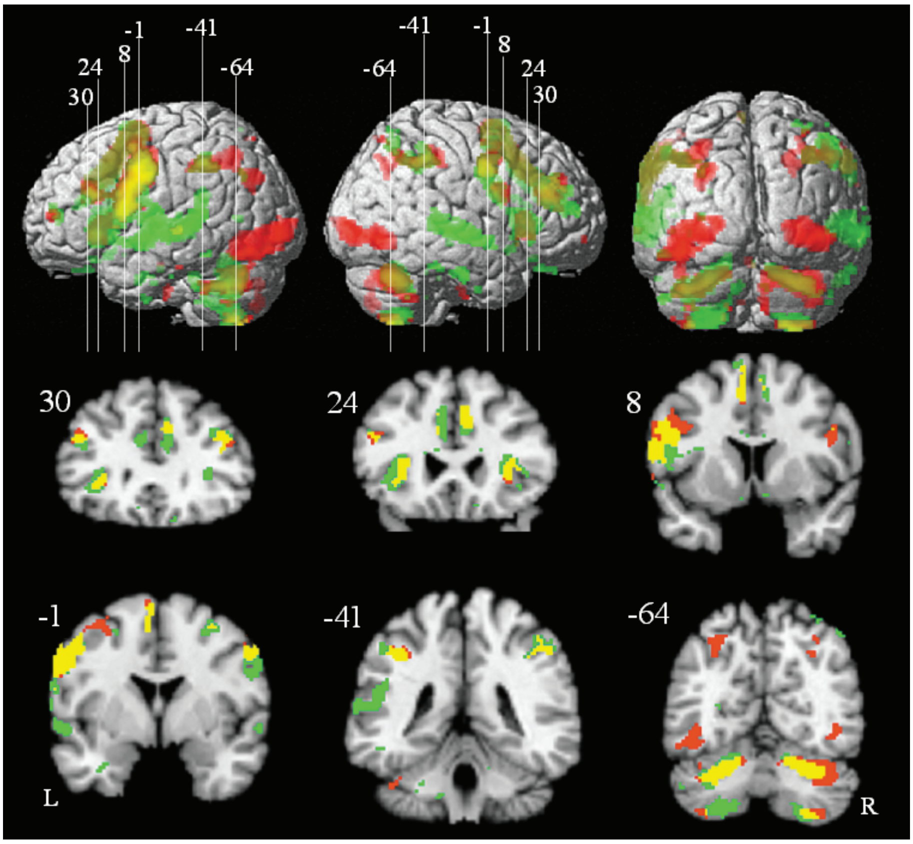

Fig. 3. Brain activation as a function of modality and working memory load. Red regions represent voxels that exceeded statistical significance threshold for the visual but not for the auditory modality; green regions represent voxels that exceeded threshold for the auditory but not visual modality; yellow regions exceeded threshold in both modalities. Voxels have been thresholded at $p < 0.001$, and all survived false discovery rate correction at $p < 0.05$. Numbers on the figure indicate distance in mm from the Y axis origin of the MNI brain.

Table 2
Visual – auditory contrast

| Hem | SPM{Z} | x | y | z | Location | Descrip | Nvox |
|---|---|---|---|---|---|---|---|
| Visual > Auditory | | | | | | | |
| Hem | SPM{Z} | x | y | z | Location | Descrip | Nvox |
| Left | 4.2 | −36 | −72 | −10 | Fus Gyr | BA 19 | 786 |
| Left | 4.1 | −40 | −82 | −2 | Inf Occ Gyr | BA 19 | |
| Left | 3.9 | −38 | −70 | −23 | Sup Cbl | Crus I | |
| Left | 3.8 | −40 | −65 | −6 | Fus Gyr | BA 19 | |
| Left | 3.8 | −36 | −79 | −13 | Fus Gyr | BA 19 | |
| Left | 3.9 | −24 | −77 | 27 | Precuneus | BA 31 | 90 |
| Left | 4.0 | −28 | −54 | 38 | Sup Par Lob | BA 7 | 70 |
| Left | 3.3 | −16 | −93 | −3 | Lingual Gyr | BA 17 | 17 |
| Left | 3.0 | −38 | −38 | −32 | Sup Cbl | Crus I | 11 |
| Right | 4.6 | 40 | −70 | −9 | Fus Gyr | BA 19 | 753 |
| Right | 4.1 | 32 | −81 | −12 | Fus Gyr | BA 19 | |
| Right | 3.4 | 28 | −60 | 39 | Sup Par Lob | BA 7 | 48 |
| Right | 3.4 | 21 | −94 | 5 | Mid Occ Gyr | BA 18 | 46 |
| Right | 3.1 | 32 | −61 | −24 | Sup Cbl | HVI | 30 |
| Auditory > Visual | | | | | | | |
| Hem | SPM{Z} | x | y | z | Location | Descrip | Nvox |
| Left | 4.8 | −57 | −23 | 10 | Sup Temp Gyr | BA 41 | 2185 |
| Left | 4.6 | −57 | −11 | 4 | Sup Temp Gyr | BA 22 | |
| Left | 4.6 | −53 | 1 | −6 | Sup Temp Gyr | BA 38 | |
| Left | 4.5 | −40 | 6 | −17 | Sup Temp Gyr | BA 38 | |
| Left | 4.5 | −60 | −7 | −1 | Sup Temp Gyr | BA 21 | |
| Left | 4.3 | −40 | −25 | 10 | Transv Temp Gyr | BA 41 | |
| Left | 4.1 | −57 | −41 | 8 | Sup Temp Gyr | BA 22 | |
| Left | 4.1 | −60 | −20 | −2 | Sup Temp Gyr | BA 21 | |
| Left | 3.4 | −12 | −53 | −12 | Sup Cbl | HIV/V | 56 |
| Left | 3.2 | −10 | 36 | 18 | Ant Cing | BA 32 | 20 |
| Left | 3.1 | −28 | −3 | −23 | Uncus | Amyg | 25 |
| Right | 5.2 | 58 | −16 | 9 | Transv Temp Gyr | BA 42 | 1395 |
| Right | 4.7 | 51 | −27 | 10 | Transv Temp Gyr | BA 41 | |
| Right | 4.7 | 62 | −19 | 4 | Sup Temp Gyr | BA 22 | |
| Right | 4.5 | 49 | −16 | −2 | Sup Temp Gyr | BA 22 | |
| Right | 3.7 | 47 | 28 | 9 | Inf Fr Gyr | BA 46 | 16 |

fusiform and inferior occipital gyri, as well as in posterior portions of the inferior parietal lobule. Additionally, activations in the inferior cerebellum appeared to be more medially distributed for the auditory condition (lobule VIII) and more lateral for visual presentation (extending into lobule VIIB). Left inferior cerebellar activation was particularly prominent for the auditory modality.

Table 2 and Fig. 4 depict regions in which auditory and visual linear load effects differed significantly in a direct comparison. The auditory modality exhibited significantly greater activation in traditional areas of auditory processing, such as the superior temporal gyri bilaterally, but also in some occipital regions including the lingual gyri and cuneus bilaterally. Additional regions of greater auditory processing were the inferior frontal and cingulate gyri bilaterally. Likewise, significantly greater visual activation was observed in fusiform and inferior occipital gyri bilaterally, as well as in left precuneus and bilateral portions of the superior parietal lobule. In the cerebellum, greater auditory processing occurred in medial portions of the cerebellar hemisphere, including lobules VIII and IX inferiorly and IV/V superiorly. Greater visual processing was observed in lateral superior cerebellar hemispheres, including lobule VI and Crus I.

## 4. Discussion

Two main conclusions regarding the modality dependence of the cerebro-cerebellar networks in VWM can be drawn from these data. The first is that, although many neocortical and cerebellar regions are utilized in both modalities (Table 1), there are important differences in brain activation between the modalities which can help us understand how modality specific information is processed in the brain. The second is that there are interesting modality specific topographical differences in cerebellar activation, with auditory presentation resulting in greater medial (especially left inferior medial) cerebellar hemisphere activations and visu-

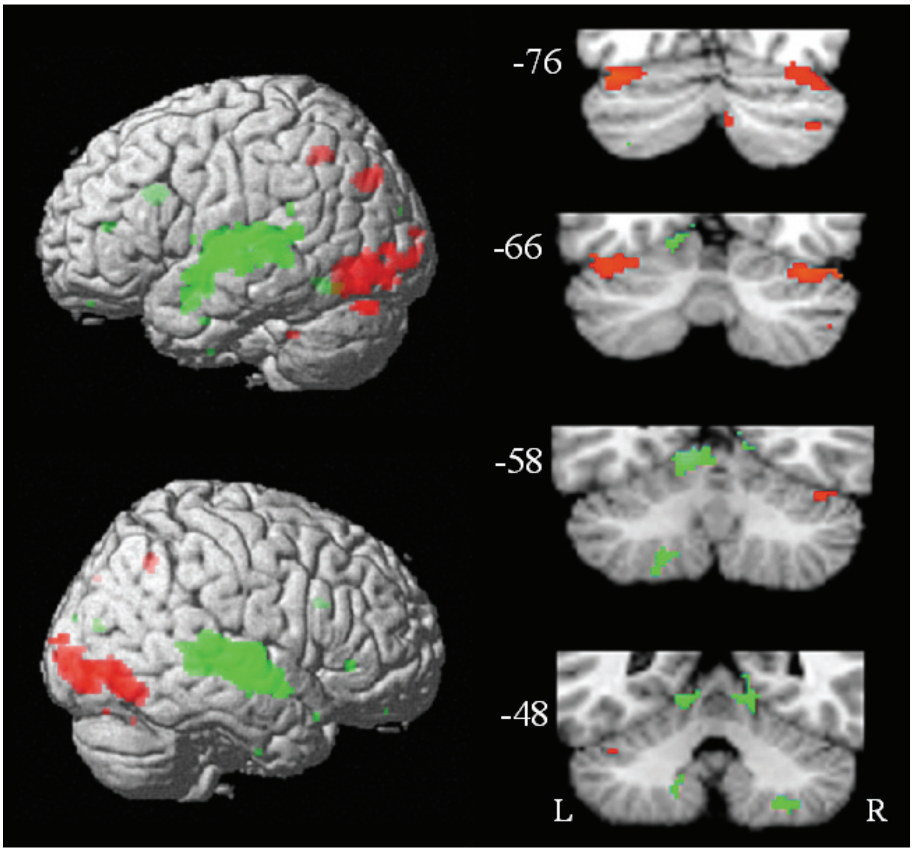

Fig. 4. Differences in activations patterns as a function of input modality. Regions where the visual Load 6 – Load 2 condition showed significantly greater activation than the comparable auditory condition are displayed in the red color scheme and regions where the auditory condition showed significantly greater activation are shown in green. Activated voxels on the surface rendering were computed by a paired t-test between the modalities and thresholded at $p < 0.005$. Although the superior cerebellar activations were significant at this threshold, the inferior cerebellar activations (which a priori were hypothesized to show greater auditory response) are depicted here at a $p < 0.025$ threshold.

al presentation resulting in greater lateral hemisphere activations. The latter result is consistent with the hypothesis that this region is involved in the rapid translation of visual material into an articulatory trajectory which requires access to the phonological loop.

Although most reports suggest an advantage for auditory stimuli during tests of memory span and working memory [8,20,40,46], we showed a slight advantage for visual stimuli (Fig. 2). The reduced accuracy with auditory compared to visual stimuli in this study probably results from the more challenging auditory environment of the scanner. In addition, because the auditory stimuli were presented as a digitized computer voice, some of the phonologically similar stimuli were difficult to differentiate. However, when subjects did answer correctly (Fig. 2), their response latencies were not significantly different. The lack of an interaction effect between modality and memory load indicates that the relative increase in reaction time and decrease in accuracy from load 2 to load 6 persists across the different modalities and that any differences in performance seen between modalities are equated over the high and low memory load conditions. Our use of a within subject design and closely equated task conditions across the two modalities, ensured that task-related activation differences arise from modality-specific effects. This behavioral result is consistent with that of Schumacher and colleagues who reported that subjects responded significantly faster on a visual 3-back task than a similar auditory task [55]. They postulated that aurally presented stimuli took longer to encode than visual stimuli. Other studies in the literature also reported an inversion of the modality effect, showing superior performance for visual stimuli [7,47].

Activated regions in our study relate to many other neuroimaging studies of VWM [1,15,23,44,49,59, 60] and included precentral/premotor (BA 6), inferior frontal (BA 44/47), parietal (BA 40), and cingulate (BA

24/31) regions, and the cerebellum. With few exceptions, all of these areas were activated in both visual and auditory modalities. In addition to yielding information about modality processing streams, these data also provide further evidence for cerebro-cerebellar involvement in VWM and human cognition [15,23,50].

Although most regions typically associated with VWM showed a high degree of overlap between modalities, a direct statistical comparison between modalities yielded several differences (Table 2). Most differences in activation were restricted to regions responsible for primary sensory processing (i.e. left and right temporal gyri for auditory and bilateral fusiform and occipital gyri for visual stimuli). However, we noted an interesting inferior-to-superior gradient of activation for aurally vs. visually encoded stimuli in two regions thought to be critically involved in phonological loop function, the left inferior parietal lobule, which has been linked to phonological storage, and the left inferior frontal region, which has been linked to the articulatory control system.

For the inferior parietal lobule, there were regions of left supramarginal gyrus that show greater activation for the auditory relative to visual condition, whereas the superior parietal lobule exhibited greater activation for the visual condition. Furthermore, looking at the single modality activations (Table 1), the common region of activation in Brodmann Area 40 (108 voxels) has a maximum in the supramarginal gyrus for the auditory condition but for the visual condition the maximum is more posterior and superior in the inferior parietal lobule. The greater activation observed during visual VWM in the left inferior parietal region is also consistent with other published reports [21] using an N-back task. These results also support claims by Vallar et al. [65] and others [46,51] that information of different modalities enters the phonological loop through different processing streams. Vallar et al. [65] discusses an anatomo-functional model of the components of phonological short-term memory in which auditory input directly accesses the phonological short-term store. Meanwhile, visual input must first undergo visual analysis and orthographic to phonologic recoding before entering the network at the level of the phonological output buffer.

In addition to the left parietal cortices, we also observed modality differences in the left frontal regions. Although these regions activated for both modalities, Table 1 indicates that within the large region of activation common to both modalities (924 voxels), peak activation for the auditory modality was observed in inferior frontal gyrus (BA 44) whereas for the visual modality the peak was in the precentral gyrus (BA 6). Auditory-specific frontal activation was also observed in BA 44 and visual-specific activation was found in BA 6 (see Fig. 3, 8 mm section). Like the inferior parietal region, these results suggest different processing streams, with auditory information entering more inferiorly to left frontal cortex than visual information. Frontal regions showed greater responses for auditory stimuli in Crottaz-Herbette et al.'s study [21], but failed to show an auditory preference in other imaging studies [55]. Studies from both Chen and Desmond [16] and Chein and Fiez [14] where verbal stimuli were presented in the visual modality demonstrated increased activation in the inferior frontal region coupled with superior cerebellar during the encoding phase of a VWM task similar to the inferior frontal gyrus activation seen for the visual modality in the present study.

The increased activation observed in the lateral superior cerebellum during visual VWM, indicates that this region might be recruited during orthographic to phonologic recoding of visual information. This result is supported by data from Chen and Desmond [16] which also demonstrates recruitment of the superior cerebellum in the encoding phase of a visual VWM task. Both Schumacher et al. [55] and Crottaz-Herbette et al. [21] reported superior cerebellar activations during VWM, and Crottaz-Herbette et al. even provided statistical evidence for greater right superior cerebellar activation (lobule VI) for visual over auditory VWM. These studies support and reinforce the role of the superior cerebellum in VWM, especially in the encoding and translation of visual information.

The right inferior cerebellum is activated during both visual and auditory VWM, which, in conjunction with neuronatomical evidence suggesting connectivity between temporal/parietal regions and the inferior cerebellum [15,53], is consistent with its role in phonological processing. The left inferior cerebellum (especially hemispheric lobule VIII) on the other hand is preferentially activated with aurally presented information. This is supported by results from a recent study from our laboratory, in which the contributions of individual cerebellar lobules to behavioral impairments were studied in children after cerebellar tumor resection, demonstrating that damage to the left inferior hemispheral lobule VIII is associated with impaired auditory digit span performance [38]. Ravizza and colleagues similarly reported impaired digit span performance for aurally-presented stimuli [50] in an adult cohort of cerebellar stroke and tumor patients, and Chiricozzi et al. [18] reported a case study of a patient with cerebellar damage to left hemispheral lobule VIII and right hemispheral lobule V that showed impaired phonological storage. Using a related task of VWM, Hayter et al. reported cerebellar cortical lobule VII activation as well as activation in similar cortical structures to the present study [30]. Stimuli in their experiment were presented aurally, however subjects were required to perform further manipulation of the items in memory, thus increasing and expanding the cognitive demand. This might explain why their lobule VII activations were localized more medially and posteriorly than the lobule VII activations found in the present study.

Several models, many already discussed above, have been proposed to explain the neuronal processing of VWM [3,5,12,23,65]. Only Vallar et al.'s model attempts to account for modality specific processing streams in VWM. Inherent in the notion of phonological recoding is the possibility that visually-presented letters are recoded into an auditory representation. Figure 3 and Table 1 indicates that a region within Brodmann area 21/22 is activated by the visual modality as well as the auditory modality, and may represent a substrate of the recoding process. Henson et al. [31] proposed a tentative mapping of the Burgess and Hitch model onto the brain which also includes modality specific inputs. In this model, auditory input is processed directly by the inferior parietal cortex. They predict inferior parietal activation in any task involving phonological recoding or rehearsal, which is consistent with our results. Visual input, on the other hand, is first processed by either the inferior frontal cortex (equivalent to the phonological output buffer in Vallar's model) or the posterior temporal cortex, before being filtered into the phonological loop. In Henson's model, the inferior frontal cortex is the only region with reciprocal connections to the inferior parietal cortex and so visual items processed in posterior temporal cortex, must first be handled by the inferior frontal cortex before gaining access to the phonological loop. Henson and colleagues [31] expect these regions to be active both in the recoding of visual items and in the rehearsal of phonological information, which is also consistent with results from this study. Although this model accounts for modality-specific input, it does not include the cerebellar contribution to the VWM circuitry.

Although the discussion above has focused on inferior parietal and frontal neocortical regions thought to subserve critical verbal working memory functions of phonological storage and articulatory control, respectively, as well as specific cerebellar regions hypothesized to interact with those neocortical regions in the inferior and superior cerebellar hemispheres, activations in other regions listed in the tables can be noted. For example, medial temporal activations were observed in the auditory condition, and such activations have been characterized in supporting working memory retrieval [42]. Activations were also observed for both modalities in the insula bilaterally. This region is consistently activated in tasks that involve making decisions about briefly encoded material, such as letters [13, 15,16,37,68], pseudowords [26], colors [17] and spatial configurations [48]. This region has also been associated with response inhibition [41] which suggests that it plays a critical role in the identification and assessment of relevant stimuli leading up to a response decision.

In summary, data from the present experiment extend our current understanding of how VWM is processed in the brain and how cerebro-cerebellar structures are organized. Inspection of Fig. 3 suggests a visual to auditory emphasis in function of the cerebellum when progressing from lateral to more medial regions (see Fig. 3, −64 mm section and Fig. 4). We speculate that this lateral to medial progression may bear some similarities to that reported by Hulsmann et al. [33]. In that study, medially-localized cerebellar activation was associated with the primary response, a finger press, whereas more lateral cerebellar activation was linked to the planning and preparation for that response. In the present experiment and in verbal working memory in general, the primary representation of information is phonological and auditory in nature. The more lateral localization of activation for visually presented stimuli may therefore represent similar preparatory processes to convert the orthographic coding of information into the primary phonological state.

## Acknowledgements

Supported by NIMH (MH60234) and Stanford Medical Scientist Training Program. We would like to thank Pam Schraedley-Desmond for statistical assistance. These data were included in Matthew Kirschen's doctoral dissertation entitled, "Cerebro-cerebellar contributions to human verbal working memory."
